# Assessing the Correlation between Grey and White Matter Damage with Motor and Cognitive Impairment in Multiple Sclerosis Patients

**DOI:** 10.1371/journal.pone.0063250

**Published:** 2013-05-16

**Authors:** Emilia Sbardella, Nikolaos Petsas, Francesca Tona, Luca Prosperini, Eytan Raz, Gianvito Pace, Carlo Pozzilli, Patrizia Pantano

**Affiliations:** 1 Department of Neurology and Psychiatry, Sapienza University, Rome, Italy; 2 Department of Psychology, Sapienza University, Rome, Italy; Institute Biomedical Research August Pi Sunyer (IDIBAPS) - Hospital Clinic of Barcelona, Spain

## Abstract

**Background:**

Multiple sclerosis (MS) is characterized by demyelinating and degenerative processes within the central nervous system. Unlike conventional MRI,new advanced imaging techniques improve pathological specificity and better highlight the relationship between anatomical damage and clinical impairment.

**Objective:**

To investigate the relationship between clinical disability and both grey (GM) and white matter (WM) regional damage in MS patients.

**Methods:**

Thirty-six relapsing remitting-MS patients and 25 sex- and age-matched controls were enrolled. All patients were clinically evaluated by the Expanded Disability Status Scale and the Multiple Sclerosis Functional Composite (MSFC) scale, which includes the 9-hole peg test (9HPT), the timed 25-feet walking test (T25FW) and the paced auditory serial addition test (PASAT). All subjects were imaged by a 3.0 T scanner: dual-echo fast spin-echo, 3DT1-weighted and diffusion-tensor imaging (DTI) sequences were acquired. Voxel-based morphometry (VBM) and tract-based spatial statistics (TBSS) analyses were run for regional GM and WM assessment, respectively. T2 lesion volumes were also calculated, by using a semi-automated technique.

**Results:**

Brain volumetric assessment of GM and DTI measures revealed significant differences between patients and controls. In patients, different measures of WM damage correlated each-other (*p*<0.0001), whereas none of them correlated with GM volume. In patients, focal GM atrophy and widespread WM damage significantly correlated with clinical measures. In particular, VBM analysis revealed a significant correlation (*p*<0.05) between GM volume and 9HPT in cerebellum and between GM volume and PASAT in orbito-frontal cortex**.** TBSS showed significant correlations between DTI metrics with 9HPT and PASAT scores in many WM bundles (*p*<0.05), including corpus callosum, internal capsule, posterior thalamic radiations, cerebral peduncles.

**Conclusions:**

Selective GM atrophy and widespread WM tracts damage are associated with functional impairment of upper-limb motion and cognition. The combined analysis of volumetric and DTI data may help to better understand structural alterations underlying physical and cognitive dysfunction in MS.

## Introduction

In patients with Multiple Sclerosis (MS), conventional Magnetic Resonance Imaging (MRI) identifies brain multifocal white matter (WM) damage, appearing as T2 hyperintense lesions (T2L), but it is often not suitable to evidence the subtle and widespread abnormalities in the so-called normal-appearing WM (NAWM) [1], [2] and in the grey matter (GM) [3], [4]. In comparison with classical MRI measures, advanced imaging techniques improve pathological specificity and better highlight the correlations between anatomical damage and clinical impairment [5], [6]. Specifically, in MS patients volumetric T1 scans and diffusion tensor imaging (DTI), both analysed by voxel-wise methods, i.e.voxel-based morphometry (VBM) and tract-based spatial statistics (TBSS), can better delineate the changes occurring in the GM and in the WM, respectively.

While VBM analysis [7] is a technique able to demonstrate regional GM atrophy [8], [9], TBSS method [10], utilizing DTI data, provides information about WM microstructural damage [11]. Four metrics are obtained by DTI: fractional anisotropy (FA), reflecting the degree of directionality within the fiber tracts; mean diffusivity (MD), representing the free water diffusion; λ1 [axial diffusivity (AD)], expression of diffusivity parallel to the fibers; λ2+λ3 [radial diffusivity (RD)], expression of diffusivity perpendicular to the fibers [5], [12]. Lower FA and higher MD are generically indicative of tissue damage, whereas changes in AD and RD have been hypothesized to differentiate respectively axonal injury from demyelination in WM tracts [13].

Several TBSS and VBM studies provided information on diffusion abnormalities and regional GM atrophy in all phenotypes of MS pathology [14]–[18], but in few cases these two approaches have been used in combination [19],[20]. Moreover, how much structural abnormalities evidenced by TBSS and VBM correlate with clinical features is still under evaluation. In particular, to the best of our knowledge, no study evaluated the correlation between VBM and TBSS analyses applied together with the clinical impairment of different functional systems in relapsing-remitting (RR) MS patients. Here, we aimed to investigate the relationship between structural WM and GM damage with disability in a cohort of RRMS subjects, by combining the results of TBSS and VBM analyses. In particular, we were interested in detecting the role of specific brain tissue damage in the impairment of motor and cognitive functions. The combined use of the two techniques offers the opportunity to detect focal WM and GM damage, which can contribute to the clinical deficits.

## Materials and Methods

### Ethics Statement

This study was approved by the Institutional Review Board of “Sapienza” University of Rome. Written informed consent was obtained from each participant before starting.

### Patients

We consecutively recruited right-handed patients aged from 18 to 50 years, diagnosed with RRMS according to the revised McDonald criteria [21] and scored with the Expanded Disability Status Scale (EDSS) [22] from 0 to 5.5. The exclusion criteria were: relapse occurring over the previous six months, first dose of disease-modifying or symptomatic treatments and any medication change in the previous three months, other significant pathologies, contraindications to MRI or poor quality of the images acquired. Patients showing one or more gadolinium-enhancing lesions (GEL) on baseline MRI were also excluded, to avoid effects of oedema and inflammation on DTI measures.

Eligible patients underwent clinical assessment and MRI in the same session. A sample of healthy subjects (HS) was used as control group.

### Clinical Assessments

For each patient, sex, age and disease duration variables were obtained. All patients were clinically evaluated by means of the EDSS and the MSFC. MSFC includes 3 subscales: the timed 25-feet walk test (T25FW), expressed in seconds spent to walk 25 feet and used to measure leg function, the higher the time the worse the walk function; the timed nine-hole peg test (9HPT) performed with dominant (9HPTD) and non-dominant (9HPTND) hand, scored in seconds spent to insert and remove nine pegs in as many holes and used to measure upper extremities function, the longer the time the worse the function; the 2- and 3-seconds (s) version of the paced auditory serial addition test (PASAT), expressed in number of correct answers and used to measure neuropsychological function, the higher the score the best the performance [23]. The T25FW, 9HPT and PASAT raw values were converted to z scores using normative data [24].

### MRI Acquisition and Analysis

Subjects were studied with a 3.0 T scanner (Verio, Siemens AG, Erlangen, Germany). The body coil was used for signal transmission, and the manufacturer 16 channel head coil designed for parallel imaging (GRAPPA) was used for signal reception. Slice orientation parallel to the subcallosal line was assured by acquiring a multi-planar T1-weighted localizer at the beginning of each MRI exam. The following sequences were acquired in a single session: 1) Dual-echo turbo spin echo [Proton Density (PD) and T2-weighted] axial sequence [repetition time (TR) = 5,310 ms, echo time (TE) = 10/103 ms, echo train length = 28, matrix = 384×90, field of view (FOV) = 220 mm^∧^2, acquisition time = 5′04″], 40 slices, 4 mm thick and 0 mm gap; 2) 3D-T1-weighted magnetization-prepared rapid acquisition gradient echo (MPRAGE) sequence with 176 axial, 1 mm slices, no gap (TR = 1,900 ms, TE = 2.3 ms, flip angle = 9°, matrix = 256×98, FOV = 240 mm^∧^2, acquisition time = 3′48″); 3) DTI acquired with an axial single-shot echo-planar spin-echo sequence with 30 gradient directions (TR = 12,200 ms, TE = 94 ms, matrix = 96×100, FOV = 250 mm^∧^2, b = 0 and 1,000 s/mm^∧^2,averages = 2, acquisition time = 13′15″), 72 slices, 2 mm thick, no gap; 4) T1-weighted spin echo after administration of gadolinium-based contrast agent (TR = 550 ms, TE = 9.8 ms, matrix = 384×90, FOV = 220 mm^∧^2, acquisition time 2′15″), 40 slices, 4 mm thick, no gap.

Image data processing was performed on a Linux workstation using Jim 5.0 software (Xinapse System, Leicester, UK; http://www.xinapse.com), the FMRIB Software Library (FSL) 4.1 package (FMRIB Image Analysis Group, Oxford, UK; http://www.fmrib.ox.ac.uk/fsl), MATLAB 7.0 (Mathworks, Natick, Massachusetts, USA) and the Statistical Parametric Mapping 8 (SPM8) software (Wellcome Department of Cognitive Neurology, London, UK; http://www.fil.ion.ucl.ac.uk/spm).

#### Lesion volumes

T2 lesion volume (T2LV) was obtained with a previously described technique [25], using a semi-automated technique based on local thresholding by the Jim software; lesions were segmented on PD images, while T2-weighted images were used to increase the confidence level in lesion identification. This procedure yielded a quantification of the lesion burden and the creation of a binary lesion mask needed for the volumetric analysis.

#### Volumetric assessment

We used the same methodology as in our previous works [19],[26]. Briefly, in patients, after the co-registration of each T2 image to the correspondent T1-3D sequence, lesion masks were used to remove lesions in T1 volumetric scans, thus avoiding their erroneous inclusion in the GM volume assessment by the segmentation output. T1-3D underwent automated segmentation in SPM8 to yield GM, WM and cerebro-spinal fluid (CSF) images. The VBM protocol consists of an iterative combination of segmentations and normalisations to produce a GM probability map. Normalized GM images were modulated, i.e. multiplied by the local value derived from the deformation field, thereby preserving within-voxel volumes that may have been altered during non-linear normalization. GM, WM and CSF volumes were recorded and used to calculate intracranial volume (ICV) as LV+GM+WM+CSF, and the brain parenchymal fraction (BPF) as (LV+GM+WM)/(LV+GM+WM+CSF). Data were smoothed using a 12-mm full width at half maximum (FWHM) Gaussian kernel.

The differences in regional GM volumes between patients and controls were assessed by two-sample *t* test, entering as confounding factors subject’s ICV, gender and age. Correlations between GM regional volumes and clinical scores were investigated by using one-sample *t* test: each clinical score was considered separately as the independent variable and patient’s ICV, gender, age and lesion volume entered as confounding factors. Statistical threshold was set at *p*<0.05 with family wise error (FWE) correction for multiple comparisons at the cluster-level.

Anatomical localization of significant clusters were obtained by Anatomical Automatic Labeling (AAL) Atlas in SPM8 Toolbox.

#### TBSS and DTI parameters

As in our previous works [19],[26], maps of FA, MD, AD, RD were computed for all subjects from the DTI, after eddy current correction and automatic brain extraction using FMRIB software library, which is part of the FSL. FA maps were fed into the TBSS tool, which is also part of the FSL. In the TBSS analysis, firstly, the FA data of all the subjects were aligned into a common space by non-linear registration and the mean FA image were created and thinned to obtain a mean FA skeleton, which represents the centres of all WM tracts common to the group; secondly, each subject’s aligned FA data were projected onto this skeleton. Similar processes were applied to MD, AD, and RD maps using the individual registration and projection vectors obtained in the FA nonlinear registration and skeletonization stages with TBSS_non_FA tool, part of FSL. In the WM analysis, lesions have been included. Results from pre-processing were fed into a voxel-wise cross-subject statistics analysis, to compare patients and HS and to identify the relationship between DTI values and MSFC subscales in the patients group. The differences in MD, FA, AD, and RD between patients and controls were assessed by unpaired *t* test, corrected for multiple comparison and adjusted for subject’s gender and age. The relationships between MD, FA, AD, and RD values and clinical measures in the patient group were investigated by linear regression: clinical values were entered in the one-sample *t* test as variable of interest, adjusted for the patient’s gender, age, and lesion volume.

The number of permutations was 5.000. The resulting statistical maps threshold was set at *p*<0.05, with correction for multiple comparisons, by using the threshold-free cluster enhancement (*tfce*) option in the randomise permutation-testing tool in FSL. Significant WM tracts were localized by using WM Atlas in FSL.

FA, MD, AD and RD values were obtained from WM tracts showing significant differences between patients and controls.

### Statistical Analysis

Statistical analysis was carried out using SPSS software, version 16.0 (SPSS, Chicago, Illinois, USA). All values are reported as mean ±standard deviation (SD) or median (range) as appropriate. Differences between groups were tested using *t*-test and the Fisher’s exact test, for continuous and categorical variables, respectively. Correlations among global MRI measures and between global MRI values and clinical scores were evaluated by univariate analysis (Pearson’s correlation coefficient) after correction for age and disease duration, and results corrected for multiple comparisons as needed.

## Results

### Demographic and Clinical Characteristics

Forty patients were recruited. Three patients were excluded because of presence of GEL on MRI and one patient because of the poor quality of the images acquired. Data from 36 patients and 25 age- and sex-matched HS were thus analysed and reported in the present study. Demographic, clinical and MRI characteristics for both groups are reported in **Table 1**. At the recruitment, thirty-two patients have been on treatment with disease modifying therapies for at least 6 months; four patients were not on treatment.

**Table 1:** Demographic, clinical and MRI characteristics for controls (n = 25) and patients (n = 36).

	Controls	Patients	*P*
**Age (years)**	31±6	34±8	NS
**Gender (F/M)**	16/9	26/10	NS
**Disease Duration** **(years)**	NA	7.4±6.1	NA
**EDSS, median** **(range)**	NA	2.5 (1–4.5)	NA
**DMT (y/n)**	NA	32/4	NA
**T2LV (mL)**	NA	7.3±8	NA
**GM (mL)**	679.5±64	636.6±52.6	**0.006**
**GM/ICV**	0.463±0.007	0.458±0.008	**0.014**
**WM (mL)**	487.7±47.7	448.3±44.6	**0.002**
**WM/ICV**	0.332±0.007	0.323±0.016	**0.002**
**CSF(mL)**	300.1±42	297.4±35.7	NS
**CSF/ICV**	0.20±0.010	0.21±0.016	**0.004**
**BPF**	0.79±0.01	0.78±0.02	**<0.001**
**FA**	0.52±0.02	0.47±0.04	**<0.001**
**MD (mm^2^/s)**	0.71±0.02×10^−3^	0.78±0.06×10^−3^	**<0.001**
**AD(mm^2^/s)**	1.25±0.026×10^−3^	1.36±0.085×10^−3^	**<0.001**
**RD(mm^2^/s)**	0.48±0.020×10^−3^	0.551±0.065×10^−3^	**<0.001**

Mean±SD are shown, otherwise indicated. Chi-square was used to test difference in gender, whereas unpaired t-test was used to test all other measures. F = female, M = male, mL = millilitre, mm = millimetres, s = second, EDSS = expanded disability status scale, DMT = disease modifying therapy, T2LV = T2 lesion volume, GM = grey matter, WM = white matter, CSF = cerebrospinal fluid, ICV = intracranial volume, BPF = brain parenchymal fraction, FA = fractional anisotropy, MD = mean diffusivity, AD = axial diffusivity, RD = radial diffusivity, NS = not significant, NA = not applicable.

### Global MRI Measures of GM and WM Damage

Global brain volumetric assessment of GM, WM and BPF, as well as GM/ICV, WM/ICV, CSF/ICV ratios and DTI measures revealed significant differences between patients and HS (**Table 1**).

Correlations were evaluated only for normalized brain volumes. The various indexes of WM damage (T2LV, FA, MD, AD, RD and WM/ICV) significantly correlated each other (*p*<0.0001), but none of them showed a correlation with GM/ICV **(Table S1)**.

The correlations between global MRI and clinical measures are shown in **Table 2**: 9HPTND was significantly correlated with most of the WM damage indexes, whereas PASAT 2 s score was correlated with GM/ICV ratio.

**Table 2:** Correlations between MRI measures of global GM and WM damage and clinical scores (EDSS and MSFC subscales).

Clinical scores	MRI measures of global GM and WM damage						
	T2LV	FA	MD	AD	RD	WM/ICV	GM/ICV
EDSS	0.328 (0.17)	0.943(−0.13)	0.457(0.13)	0.167(0.24)	0.590(0.09)	0.370(−0.16)	0.649(0.08)
9HPTD	0.117(0.27)	0.015(−0.41)	0.041(0.35)	0.270(0.19)	0.022(0.39)	0.34(−0.17)	0.675(−0.08)
9HPTND	**0.001(0.55)**	0.029(−0.41)	**0.003(0.49)**	**0.003(0.49)**	**0.004(0.48)**	0.012(−0.43)	0.220(−0.22)
PASAT 2 s	0.368(−0.16)	0.189(0.23)	0.235(−0.21)	0.480(−0.12)	0.218(−0.22)	0.798(−0.05)	**0.005(0.47)**
PASAT 3 s	0.075(−0.31)	0.207(0.22)	0.300(−0.18)	0.492(−0.12)	0.253(−0.20)	0.961(−0.01)	0.309(0.18)
T25FW	0.543(−0.11)	0.891(0.02)	0.906(0.02)	0.774(0.05)	0.951(0.01)	0.611(0.09)	0.147(−0.25)

Results are reported as *p*(r); ratios between GM/ICV and WM/ICV have been considered.

T25FW = timed 25-foot walk test, 9HPT = nine-hole peg test performed with dominant (D) and non-dominant (ND) hand, PASAT = paced auditory serial addition test at 2 and 3 seconds,

EDSS = expanded disability status scale, T2LV = T2 lesion volume, GM = grey matter, WM = white matter, ICV = intracranial volume, FA = fractional anisotropy, MD = mean diffusivity, AD = axial diffusivity, RD = radial diffusivity. Results from Pearson’s correlation coefficient. After correction for multiple comparison, *p*<0.007 was considered as significant.

### Regional GM Volumetric Assessment and VBM

**Figure 1:**
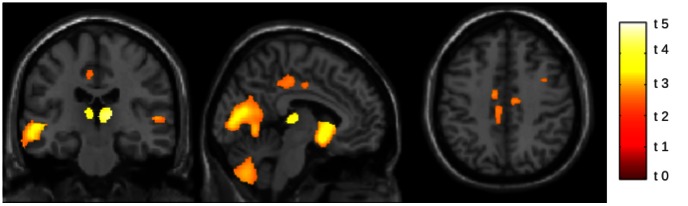
Differences in GM volume between patients and controls. GM volume is decreased in patients group in comparison to controls. Differences are evident in the cerebellum, thalamus, subgenual gyrus, middle cingulate cortex, superior frontal gyrus, occipital and temporal cortices, bilaterally. Clusters of significant differences are superimposed on sagittal, coronal and axial slices of the single-subject T1 template provided with SPM8. (VBM analysis, SPM 8, two-sample, *p*<0.05, FWE corrected; t value are shown).

Regional VBM analysis revealed clusters of reduced GM volume in patients with respect to HS in the cerebellum, thalamus, subgenual gyrus and middle cingulate cortex, superior frontal gyrus, occipital and temporal cortices bilaterally (**Figure 1**). No cluster of increased GM volume was found in patients with respect to HS.

**Figure 2:**
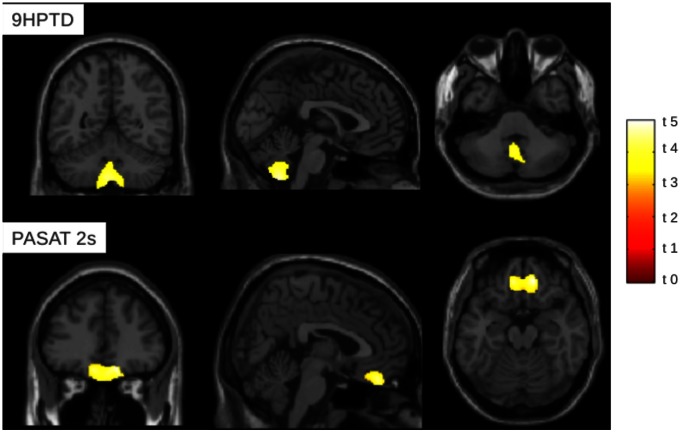
Correlation between GM volume in patients and MSFC subscales. On the top of the figure, GM volume negatively correlates with 9HPTD score at cerebellum level. On the bottom of the figure, GM volume positively correlates with PASAT 2 s at level of orbitofrontal cortex. Clusters of significant correlations are superimposed on sagittal, coronal and axial slices of the single-subject T1 template provided with SPM8. (VBM analysis, SPM 8, one-sample, *p*<0.05, FWE corrected; t values are shown).

Correlations between regional GM volumes and clinical MSFC scores showed a significant inverse correlation between GM volume in the cerebellum (lobules and vermis VIII and IX bilaterally, crus 1 on the left) and the performance at 9HPTD **(** **Figure 2** **)**; a significant positive correlation was also found between GM volume in the orbito-frontal cortex, bilaterally, and the PASAT 2s score **(** **Figure 2** **)**.

### DTI Parameters and TBSS

**Figure 3:**
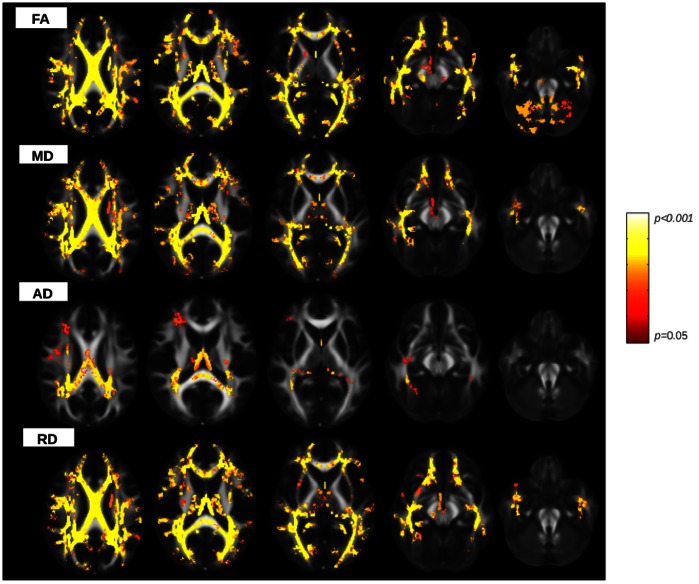
Differences in DTI measures between patients and controls. Statistical maps showing voxels which exhibit differences in DTI parameters in patients versus controls (red and yellow colours, according to the lower and higher significance, respectively ). FA is significantly decreased and MD, AD, RD are increased in patients group in comparison to controls. Differences are widespread and evident in the corona radiata, corpus callosum, internal and external capsule, superior cerebellar peduncles, superior and inferior longitudinal fasciculum, posterior thalamic radiations and cerebral peduncle bilaterally. All WM tracts are overlaid on MNI152 1 mm standard image.(TBSS analysis, two-sample, *p*<0.05, *tfce* corrected).

When patients were compared with HS, TBSS showed widespread significant differences in the FA, MD, RD pattern across all over the skeleton, including corpus callosum (CC), corona radiata (CR), superior and inferior longitudinal fasciculum (SLF and ILF), internal and external capsule, posterior thalamic radiations, cerebral peduncles, superior cerebellar peduncles bilaterally (**Figure 3**). AD was significantly different between patients and HC, but showed a less pronounced and circumscribed involvement of WM tracts in comparison to the other DTI metrics.

**Figure 4:**
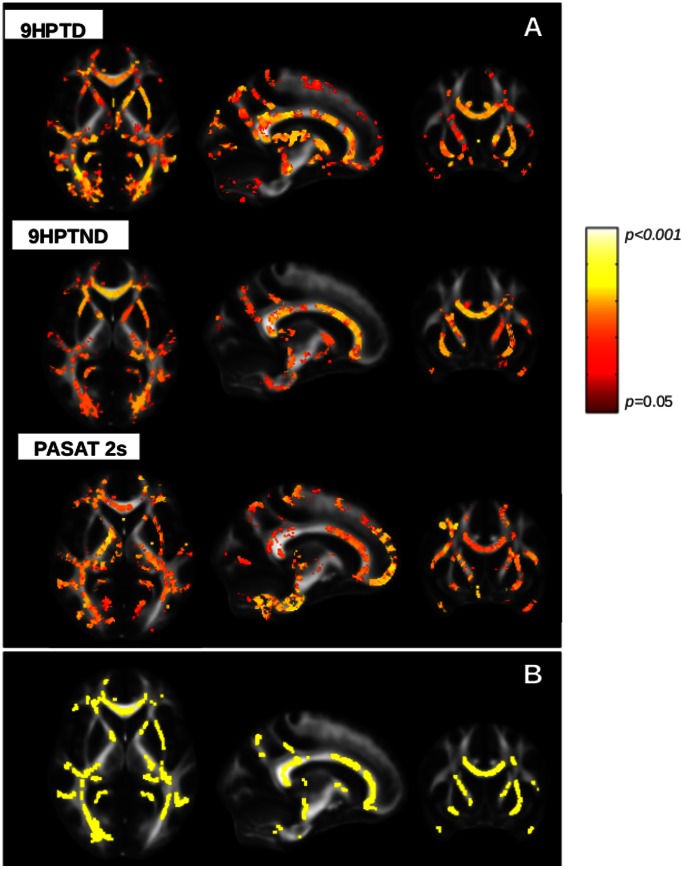
Correlation between FA in patients and MSFC subscales. Statistical map showing voxels which correlate with clinical scores. **a)** FA negatively correlates with 9HPT score obtained with dominant and non-dominant hand in corpus callosum, internal and external capsule, posterior thalamic radiations, cerebral peduncles. FA is positively correlated with the PASAT score at 2 seconds in the internal and external capsule, corpus callosum, posterior thalamic radiations, cerebral peduncles (red and yellow colours according to the lower and higher significance, respectively). **b)** WM tracts with reduced FA that are common to the WM skeletons resulted by the correlation between FA values and 9HPTD, 9HPTND and PASAT 2 s. All WM tracts are overlaid on MNI152 1 mm standard image. (TBSS analysis, one-sample, *p*<0.05, *tfce* corrected).

In the patient group, we obtained maps of significant negative correlation between FA values and the 9HPTD and 9HPTND scores and maps of significant positive correlation between FA values and the PASAT 2s scores in many WM bundles (**Figure 4a**). These maps of correlation had in common the WM tracts with decreased FA at level of CC, internal and external capsule, posterior thalamic radiations and cerebral peduncles (**Figure 4b**). MD values positively correlated with the 9HPTD and 9HPTND scores in the CC and posterior CR bilaterally, and with PASAT 2 s in the right ILF, right cerebral peduncle and left cingulum. AD and RD showed a positive correlation with the 9HPTND in the same areas as MD. No further significant correlation was found.

## Discussion

This study combines a non-hypothesis-driven whole-brain GM and WM analysis [7],[10] with a double aim: firstly to investigate a cohort of RRMS patients for the presence of brain microstructural abnormalities when compared to HS; secondly to detect the correlation between brain damage location and clinical performances in patients. The novelty of our work is the combined use of two new MRI techniques, evaluating different aspects of structural brain damage. Their contemporary application may provide complementary information useful to understand the pathological damage underlying clinical symptoms.

Our study confirms abnormalities involving both WM and GM in patients when compared to controls. Regarding the WM damage, significant changes were observed in the whole WM volume, as well as in global DTI parameters and TBSS analysis, thus supporting previous studies [5],[12],[19],[27]. In the most of the WM fiber bundles, reduced FA was associated to increased MD and RD, as a result of demyelination processes [13]. On the other hand, only in some tracts of these bundles, an increased AD has been also found, likely as a consequence of axonal loss processes [13],[14],[28]. Our cohort of patients was not heavily disabled and the more widespread involvement of WM tracts alterations in terms of FA, MD and RD in comparison to AD suggests the prominence of demyelinating rather than degenerative processes in the WM of these subjects.

As regards to the GM damage, global GM volumes resulted significantly lower in patients than in controls, whereas regional analysis evidenced a significant atrophy involving the cerebellum, thalamus, cingulum, occipital and temporal cortices bilaterally in MS. It is well known that atrophy occurs since the earliest phase of the disease, both at subcortical [26],[29],[30] and cortical level [31]–[33], and these findings would strengthen the existence of a primitive GM involvement that may occur independently from the WM damage.

A significant correlation was found among measures of WM damage, i.e DTI parameters, WM/ICV ratio and T2LV, thus suggesting that MS microstructural tissue abnormalities are partially related to the macroscopic focal lesions detectable with conventional MRI techniques [34]. On the other hand, no relationship emerged between measures of WM damage and GM/ICV ratio. Even though a relationship between WM lesions and GM damage has been demonstrated [32],[33], our results would support the more recent hypothesis of the partial independence of pathological processes affecting WM and GM both at early and later stages [26],[35],[36]. Further, with the progression of the disease course, GM atrophy development is greater than WM damage [37]. The relatively long disease duration of some patients in our cohort might have been responsible for the failure to detect a relationship between the two measures. Another possible explanation, might be related to our sample size, underpowered to detect a possible correlation.

When we investigated the relationship between global MRI measures and clinical scores (i.e. EDSS and MSFC subscales), hand dexterity significantly correlated with almost all the WM metrics, whereas cognitive performance correlated with the GM/ICV ratio. Specifically, test performed with the non-dominant hand, was strictly linked to the widespread WM damage, whereas the PASAT score at 2 s correlated with the GM damage. The findings strengthened the relevance of WM damage and GM atrophy in causing motor and cognitive impairment, respectively. Previous works found correlations between hand dexterity and WM damage [5],[15] and between PASAT score and GM [38],[39]. However, we did find a correlation particularly with the clinical tests performed at the highest level of difficulty, i.e. 9HPTND and PASAT 2 s. A possible explanation would be that more complex tests might require more attention on executive functioning and temporal sequencing [40] and might need the integrity of connections of several areas. Accordingly, these tests may result more sensitive than others to clinically reflect subtle structural damage visible on MRI in that patients not severely clinically impaired, as the case of our cohort.

When we analysed the correlations between regional GM and WM damage and clinical scores, we found that circumscribed GM abnormalities and diffuse WM tracts alterations correlated with upper limb and cognitive dysfunctions. We did not identify regions of anatomical correspondence between the location of reduced FA values and GM atrophy in patients. This finding is not in agreement with the results of Bodini et al., who studied patients with secondary progressive MS and found a relationship between the topographical location of WM and GM damage [20]. A possible explanation for this discrepancy might be related to the partially different pathological events occurring in different MS phenotypes. Specifically, in secondary progressive MS patients, the causes of GM damage might be related to axonal degeneration in a greater extent than in RRMS patients [18]. The correlation between different clinical scores and similar widespread, not circumscribed, regional WM tracts might be explained by the fact that several clinical impairments may arise from damage in combined WM areas of the brain. Indeed, the tests we administered required simultaneously different kinds of abilities, i.e. hand strength and accuracy, visual acuity and attention for the 9HPT, and therefore a good performance needed the integrity of different WM tracts. However, our results are in line with previous studies, that demonstrated the correlation between DTI alterations, in the particularly in the CST and CC, with motor [41]–[43]and cognitive disability [27],[44],[45].

We found that the bilateral orbito-frontal volumes and a widespread WM damage were correlated with the performance at PASAT 2 s. Although the relationship between the PASAT score and the frontal lobe damage has been documented [38], the specific involvement of the orbito-frontal cortex is a novel finding. Recently, patients with lesions confined in the orbito-frontal cortex were clinically evaluated by using cognitive tests. Results demonstrated that orbito-frontal damage was associated with working memory maintenance, manipulation and monitoring processes [46], thus supporting our findings.

On the other hand, the cerebellar volume and a diffuse WM tracts damage were correlated with the 9HPTD score. Since the 9HPT score is typically altered in patients with cerebellar lesions [47], this correlation is in agreement with previous data [30],[48]. Indeed, a relationship between cerebellar volumes and clinical dysfunction detected by the 9HPT has emerged in most of the MS phenotypes [30],[48]. In more details, we found a correlation between the 9HPT with lobules VIII-IX and left crus I. Lobule VIII is a region linked to the sensori-motor tasks, as it presents the sensorimotor homunculi and receives sensorimotor projections [49], whereas lobule IX is usually considered essential for the visual guidance of movement [50] and it is also activated by tactile stimulation [51]. On the other hand, crus I is activated by executive function and emotional processes [49], and is thought to be involved in higher-level processes, such as spatial transformation tasks [52], that are usually lateralized to the left cerebellar hemisphere [53].

We did not find any correlation between DTI metrics and lower limb motor impairment, as scored by the T25FW. Unlike 9HPT, measuring hand dexterity, a subtle motor deficit [54] that is present even in the earliest stages of the disease [55], the T25FW is affected by severe impairment in walking, a symptom not yet detectable in our population and often related to the spinal cord damage, not evaluated in our study.

Finally, the lack of correlation between EDSS and MRI measures is not surprising [56]. EDSS is an ordinal scale with a narrow range and strongly influenced by the ambulation ability, while MSFC offers scales with wider ranges, complementing information derived by EDSS and thus resulting more suitable for clinical studies, in order to obtain a sensible clinical reflex of the concomitant anatomical damage.

### Limits of the Study

The first limit of the study is the lack of spinal cord MRI acquisition, that might have accounted for the failure of correlation between MRI and T25FW score. The second limit is that 7 of the patients, before the inclusion, have already performed once the PASAT for cognitive evaluation; we cannot completely rule out that this may have affected the results. The third limit could be represented by the inclusion of T2 lesions in DTI analysis; there is not a complete agreement on this topic, but we preferred to take into account the whole WM, including abnormalities.

### Conclusions

Overall, our findings would suggest that GM atrophy, selectively affecting different brain regions, is strictly connected with impaired cognitive and motor performances, whereas a widespread WM damage underlies different clinical deficits, i.e. some fibre bundles are equally affected in condition of either cognitive or motor disability. The combined use of new MRI analysis techniques, contemporarily assessing different aspects of brain structural damage, provides complementary information, otherwise undetectable, on the presence of concomitant pathological substrates underlying the same clinical symptom. The use of different techniques better depicts the complex structural damage responsible for the clinical impairment, thus providing the basis for the treatment and rehabilitation fields.

## Supporting Information

Table S1 **Correlations between global GM/ICV, WM/ICV and DTI measures in patients.**
(DOCX)
