# Supplementary material for: Assessing the Correlation between Grey and White Matter Damage with Motor and Cognitive Impairment in Multiple Sclerosis Patients
Source: PLoS One. 2013 May 16;8(5):e63250. doi: 10.1371/journal.pone.0063250 (PMC3655958; doi:10.1371/journal.pone.0063250)
Supplement: Table S1 — Correlations between global GM/ICV, WM/ICV and DTI measures in patients. (DOCX) [file pone.0063250.s001.docx]

**Tab S3**. Correlations between global GM/ICV, WM/ICV and DTI measures in patients.

| **MRI measures** | **MRI measures of global GM and WM damage** | | | | | | |
| --- | --- | --- | --- | --- | --- | --- | --- |
|  | T2LV | FA | MD | AD | RD | WM/ICV | GM/ICV |
| T2LV |  | 0.0001(-0.6) | 0.0001(0.71) | 0.0001(0.63) | 0.0001(0.7) | 0.0001(-0.69) | 0.13(-0.26) |
| FA | 0.0001(-0.6) |  | 0.0001(-0.92) | 0.0001(-0.75) | 0.0001(-0.96) | 0.0001(0.66) | 0.28(0.11) |
| MD | 0.0001(0.71) | 0.0001(-0.92) |  | 0.0001(0.89) | 0.0001(0.99) | 0.0001(-0.74) | 0.036(-0.36) |
| AD | 0.0001(0.63) | 0.0001(-0.65) | 0.0001(0.89) |  | 0.0001(0.85) | 0.0001(-0.80) | 0.035(-0.36) |
| RD | 0.0001(0.7) | 0.0001(-0.96) | 0.0001(0.99) | 0.0001(0.85) |  | 0.0001(-0.73) | 0.046(-0.34) |
| WM/ICV | 0.0001(-0.69) | 0.0001(0.66) | 0.0001(-0.74) | 0.0001(-0.80) | 0.0001(-0.73) |  | 0.17 (0.24) |
| GM/ICV | 0.13(-0.26) | 0.28(0.11) | 0.036(-0.36) | 0.035(-0.36) | 0.046(-0.34) | 0.17 (0.24) |  |

Results are reported as *p*(r). T2LV= T2 lesion volume, GM= grey matter, WM= white matter, ICV=intracranial volume, FA= fractional anisotropy, MD=mean diffusivity, AD=axial diffusivity, RD=radial diffusivity. Results from Pearson’s correlation coefficient, age and disease duration corrected. After correction for multiple comparison, *p<0.008* was considered as significant.
